# Facteurs associés à la tuberculose chez l'enfant au Centre Hospitalier Universitaire Mère-Enfant de Tsaralalàna, Antananarivo: une étude cas-témoins

**DOI:** 10.11604/pamj.2014.19.224.4676

**Published:** 2014-10-29

**Authors:** Fidiniaina Mamy Randriatsarafara, Barbara Elyan Edwige Vololonarivelo, Nambinina Nirina Gaby Rabemananjara, Jean Baptiste Olivier Randrianasolo, Jean de Dieu Marie Rakotomanga, Vahiniarison Dieudonné Randrianarimanana

**Affiliations:** 1 Service Technique de la Direction Centrale du Service de Santé Militaire B.P 10 Ampahibe Antananarivo; 2 Département de Santé publique, Faculté de médecine, Université d'Antananarivo; 3 Département Mère-Enfant, Université d'Antananarivo; 4 Institut National de Santé Publique et Communautaire, B.P 146 Befelatanana Antananarivo

**Keywords:** Facteurs de risque, Madagascar, pédiatrie, tuberculose, risk factors, Madagascar, pediatry, tuberculosis

## Abstract

**Introduction:**

A Madagascar, la tuberculose reste un problème de santé publique majeur, l'incidence s’élevant à 16% depuis 2009. Le présent travail a pour objet d'identifier les facteurs de risque de tuberculose chez l'enfant.

**Méthodes:**

Nous avons mené une étude rétrospective de type cas-témoins sur les facteurs de risque de la tuberculose chez les enfants de 0 à 15 ans au Centre Hospitalier Universitaire Mère-Enfant de Tsaralalàna, de Janvier 2009 à Décembre 2011. Les enfants diagnostiqués de tuberculose dont le diagnostic a été retenu par des éléments de certitude ou par le score pédiatrique de la tuberculose représentent les cas. Les enfants hospitalisés durant la même période, non tuberculeux et de même âge, sont classés témoins. L'Odds Ratio quantifie les associations.

**Résultats:**

Au total, 91 cas et 173 témoins ont été inclus. Parmi les cas, 73,62% présentent une malnutrition. De fortes associations sont démontrées avec: la malnutrition sévère (OR=6 (IC_95%_ 2,43-15,61 (p<10^-5^))); le contage tuberculeux (OR=4,71 (IC_95%_ 1,76-12,7 (p=0,003))); la non vaccination par le BCG (OR=4,21 (IC_95%_ 1,99-8,99 (p < 2.10^-5^))); le niveau intellectuel maternel bas (OR=4,17 (IC_95%_ 0,67-28,14 (p=0,06))); la taille de la fratrie à partir de 5 (OR=4,5). Des associations faibles sont retrouvées pour les autres facteurs étudiés. Les cas présentent une létalité de 18,7% contre 6,3% chez les témoins (p<10^-5^); 64,7 % des décès sont dus aux formes méningées.

**Conclusion:**

La tuberculose reste un fléau chez les enfants, avec une lourde responsabilité de la pauvreté rassemblant presque tous les facteurs sus-cités.

## Introduction

La tuberculose est due au *Mycobacterium tuberculosis*, appelé aussi « bacille de Koch » [1]. Malgré de nombreux efforts déployés par différentes nations et organismes internationaux dans la lutte contre cette maladie, elle figure toujours parmi les priorités de la santé publique, surtout dans les pays en voie développement (Countdown to 2015). D'après l'OMS, chaque année, on compte 9 millions de nouveaux cas et près de deux millions de personnes décèdent dans le monde [2, 3]. La plupart des cas se produisent dans les pays à bas niveau socio-économique dont 30% en Afrique [4]. L´ampleur de la tuberculose chez l´enfant est inconnue mais on estime qu´elle représente environ 6% de tous les cas incidents, la majorité d´entre eux survenant dans des pays à forte charge de morbidité tuberculeuse [5]. A Madagascar, l'incidence s’élève à 248 pour 100 000 habitants en 2006; 22 812 cas ont été déclarés en 2008 selon la 4^ème^ édition du manuel du programme national de lutte contre la tuberculose. Ainsi, la tuberculose sévit à Madagascar et peut toucher toutes les tranches d’âge [6]. En outre, plusieurs facteurs entrent en jeu dans la survenue de la tuberculose [7]. Une contamination se faisant le plus souvent à partir de l'adulte, les arguments cliniques et paracliniques seront rassemblés devant un contexte évocateur afin d’étayer le diagnostic [8, 9]. Les enfants peuvent être atteints de tuberculose à tout âge, mais ils le sont le plus souvent entre 1 et 4 ans [5], d'autant plus qu'ils n'ont pas été vaccinés [8]. Sans oublier la précarité et la malnutrition qui prend le poids dans le pays, les enfants n'en sont toujours pas épargnés [7, 10].

Au total, parmi les nombreux problèmes auxquels se heurte l´évaluation de la charge de tuberculose chez l´enfant, on peut citer la difficulté d´établir un diagnostic définitif avec des preuves bactériologiques pas toujours possibles [11], la présence d´une maladie extrapulmonaire, une priorité moindre pour la santé publique, et des liens insuffisants entre les pédiatres du secteur privé et le programme national de lutte contre la tuberculose [5]. Pour mieux cerner ce problème, il serait nécessaire de relever les différents facteurs de risque et de les évaluer. Le présent travail a pour objet d´identifier les facteurs de risque de tuberculose chez l'enfant dans le but de mieux cibler la lutte contre cette pathologie.

## Méthodes

### Matériels

L’étude a été menée durant une période de 3 ans, allant du mois de Janvier 2009 à Décembre 2011. Elle est basée sur la compulsion des dossiers médicaux des malades rédigés à l'admission par les médecins et internes de garde, avec les fiches contenant les résultats des examens complémentaires, les feuilles de traitement et de suivi. Les dossiers incomplets ont été exclus: 264 dossiers ont été retenus au lieu des 270 prévus.

### Patients et methods

L’étude consiste en une étude analytique rétrospective de type CAS-TEMOINS. Ont été inclus comme « cas » les enfants hospitalisés durant la période d’étude, diagnostiqués de tuberculose et déjà sous traitement, disposant d'observations médicales complètes. Les « témoins » choisis sont représentés par les enfants hospitalisés pour une raison autre que la tuberculose durant la période d’étude, dont l’âge et le sexe sont similaires à ceux des cas. Tous les cas ont été pris en compte durant la période d’étude. Pour chaque cas, deux témoins ont été pris. Au total, l’échantillon se compose de 90 cas et de 173 témoins.

#### Variables étudiées

Les variables suivantes constituent les variables dépendantes: le statut «tuberculeux» du cas par le score pédiatrique, devant être supérieur ou égal à 7 [12], le motif d'hospitalisation du témoin.

Les variables indépendantes sont représentées par: l’âge et le sexe; l’état nutritionnel, évalué selon les indices poids/taille et/ou poids/âge; le statut vaccinal de l'enfant par le BCG; le niveau socio-économique, évalué par la profession des parents; le niveau d'instruction de la mère; la taille de la fratrie; la notion de promiscuité, de contage tuberculeux, et de tabagisme passif.

Pour les cas, les formes cliniques de la tuberculose sont définies par la primo-infection tuberculeuse, la tuberculose pulmonaire, la méningite tuberculeuse, la miliaire tuberculeuse, la tuberculose des séreuses, la tuberculose ganglionnaire, ainsi que les autres localisations oto-rhino-laryngées et osseuses. La notion de fièvre est évaluée par la prise de la température axillaire, et l’état nutritionnel selon les nouvelles normes “Z score” de l'OMS [13] et le périmètre brachial.

#### Analyse statistique et mode de collectes des données

Les données ont été recueillies sur des fiches préétablies sous forme de tableaux, puis transférées et analysées sous EPI INFO™ 3.5.3 2011. Les représentations (tableaux et graphiques) sont traitées sous Word et Excel 2007. L'Odds Ratio (OR) quantifie les associations et le seuil de significativité est fixé à 0,05.

#### Ethique et confidentialité

Cette étude a été faite après l'accord du Chef de l’établissement du Centre Hospitalier Universitaire Mère-enfant Tsaralalàna. Les données saisies resteront anonymes, la confidentialité et les secrets médicaux seront respectés.

## Résultats

### Description de l’échantillon

**Figure 1:**
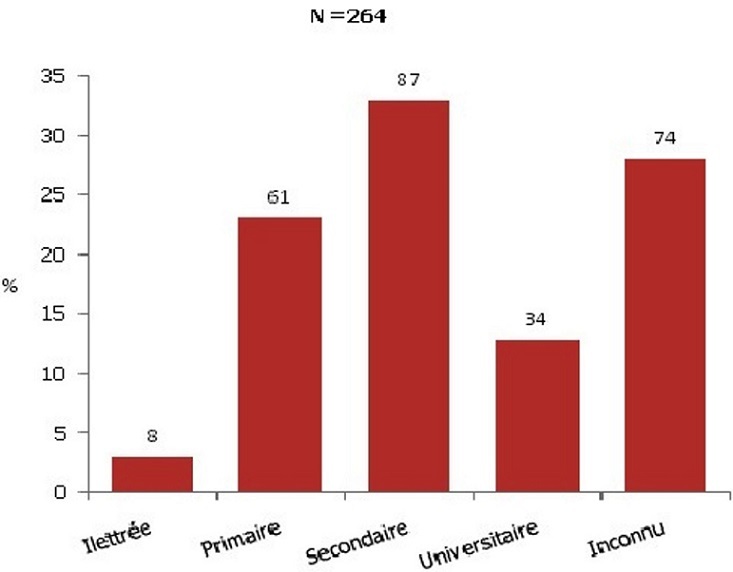
Répartition des enfants selon le niveau d'instruction de la mere

**Figure 2:**
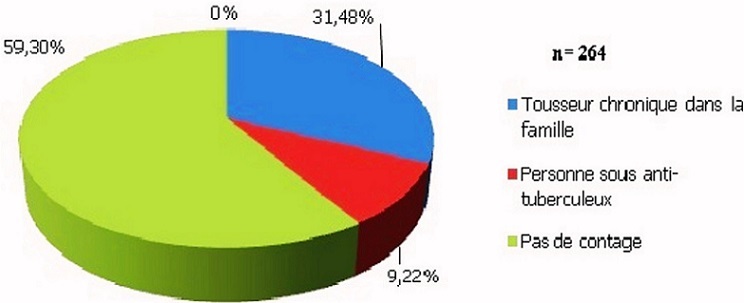
Répartition des enfants selon l'existence et le type de contage

Les enfants ont un âge moyen est de 34,34 mois; l’âge médian est de 23 mois avec une étendue de 2 à 146 mois. Le genre masculin prédomine (sex ratio: 1,13). La malnutrition sévère touche presque 1 enfant sur 3 (31,50%). Le taux de vaccination par le BCG s’élève à 84,50%. Concernant le niveau d'instruction de la mère, le niveau secondaire est le plus fréquent (Figure 1). Quant à la situation professionnelle des parents, pour la plupart des familles, seul un parent est salarié (46,21%) ou les parents sont au chômage (17,30%). Les fratries de 1 à 2 enfants sont les plus nombreuses; les fratries de 6 et de 7 représentent 3,1% de la population d’étude. Définie comme le partage par 4 personnes ou plus d'une même pièce, la promiscuité est présente chez 43,20% des familles. Parmi les antécédents familiaux, on trouve 31,40% de notion de toux chronique; seulement 9,22% des enfants vivent avec une personne sous-traitement anti-tuberculeux et 59,30% ne présentent pas de contage tuberculeux (Figure 2). Le tabagisme passif est présent chez 50,40% de la population étudiée.

L'aspect clinique et les examens de présomption concernant les cas

#### Résultats de l'examen Clinique

La majorité des enfants tuberculeux sont fébriles (89,01%), l'amaigrissement est présente chez 73,62% des enfants tuberculeux. Les signes auscultatoires et les signes neurologiques sont les plus apparents parmi les signes physiques (Tableau 1).

**Tableau 1:** Les principaux signes présentés par les enfants tuberculeux. Centre Hospitalier Universitaire Mère Enfant Tsaralalàna, 2009-2011

Signe	Effectif	Proportion (%)
Symptôme		
Fièvre	81	89,01
Toux	34	37,36
Dyspnée	23	25,27
Convulsion	22	24,17
Anorexie	10	10,98
Signes physiques		
Adénopathies	4	4,39
Signes auscultatoires	35	38,46
Hépatomegalie	6	6,59
Splénomegalie	1	1,10
Signes neurologiques	21	23,07
Autres signes	8	8,79

#### Les formes cliniques

**Figure 3:**
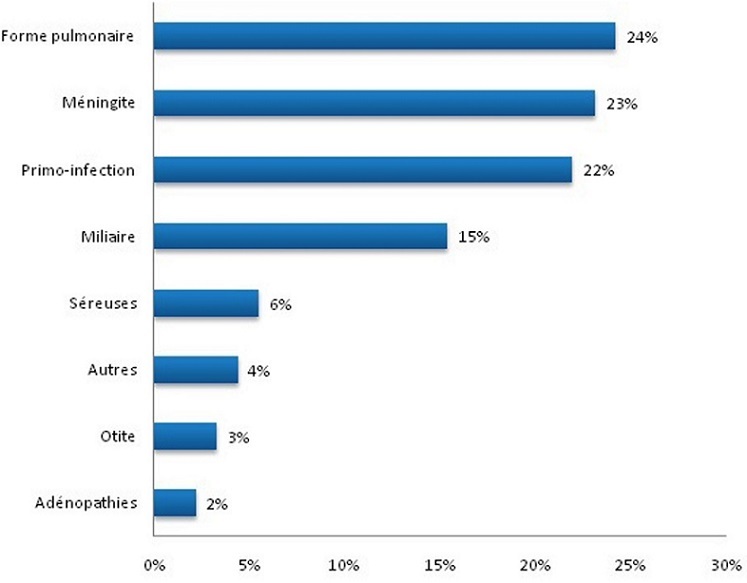
Répartition des enfants selon la forme clinique de tuberculose

Presque toutes les formes sont retrouvées mais on voit la nette prédominance des formes pulmonaires, méningites et primo-infections tuberculeuses (Figure 3).

### Répartition des cas et des témoins selon les facteurs associés à la tuberculose de l'enfant

#### Statut de l'enfant Tableau 2, Tableau 3

Les enfants âgés de 7 à 59 mois sont les plus susceptibles de contracter la tuberculose. Au-delà de 60 mois, ils en sont moins concernés. Par rapport à l’état nutritionnel, le risque de développer la maladie est proportionnellement plus élevé en s’écartant de la normale: la malnutrition sévère est significativement associée à la maladie avec six fois plus de risque que les enfants présentant un état nutritionnel normal. En outre, le risque de devenir tuberculeux est quatre fois plus élevé chez les enfants non vaccinés (p<2.10^-5^). Le niveau d'instruction de la mère est inversement associé au risque d'avoir des enfants tuberculeux. En se référant au niveau d'instruction primaire de la mère, les enfants issus de mères illettrées ont quatre fois plus de risque de contracter la tuberculose. Les enfants des parents chômeurs ont deux à trois fois plus de risque de tuberculose par rapport aux enfants qui ont des parents salariés (p = 0,01). La taille de la fratrie est significativement associée aux cas. En effet, ceux qui sont cinq ou plus dans la fratrie ont presque cinq fois plus de risque de tuberculose par rapport à ceux qui sont uniques ou deux. (p=0,0003).

**Tableau 2:** Répartition des cas et des témoins selon l’âge, l’état nutritionnel et l’état vaccinal de l'enfant. Centre Hospitalier Universitaire Mère Enfant Tsaralalàna, 2009-2011

Facteur	Cas n (%)	Témoin n (%)	Total n (%)	OR	IC_95%_
***Âge (mois)***					
0-6	25 (33,78)	49 (66,22)	74 (100)	1	
7-23	33 (35,10)	61 (64,90)	94 (100)	1,10	(0,50-2,45)
24-59	22 (36,66)	38 (63,34)	60 (100)	1,16	(0,46-2,67)
60 et plus	11 (30,55)	25 (69,45)	36 (100)	0,41	(0,08-1,77)
***Etat nutritionnel***					
Normal	24 (19,83)	97 (80,17)	121 (100)	1	
Malnutrition modérée	18 (30,00)	42 (70,00)	60 (100)	1,81	(0,67-3,91)
Malnutrition sévère	49 (59,04)	34 (40,96)	83 (100)	6,08	(2,43-15,61) *(p<10^-5^)*
***Etat vaccinal***					
Vacciné	65 (29,15)	158 (70,85)	223 (100)	1	
Non vacciné	26 (63,41)	15 (36,59)	41 (100)	4,21	(1,99-8,99) *(p < 2.10^-5^)*

**Tableau 3:** Répartition des cas et des témoins selon les conditions familiales de l'enfant. Centre Hospitalier Universitaire Mère Enfant Tsaralalàna, 2009-2011

Facteur	Cas n (%)	Témoin n (%)	Total n (%)	OR	IC_95%_
***Niveau d'instruction de la mère***					
Illettrée	5 (62,50)	3 (37,50)	8 (100)	4,17	(0,67-28,14) *(p = 0,06)*
Primaire	33 (54,09)	28 (45,91)	61 (100)	3,06	(1,15-8,25) *(p = 0,01)*
Secondaire	32 (36,78)	55 (63,22)	87 (100)	1,45	
Universitaire	11 (32,35)	23 (67,65)	34 (100)	1	
Inconnu	10 (13,51)	64 (86,49)	74 (100)	1,99	
***Situation professionnelle des parents***					
2 parents salariés	24 (25,26)	71 (74,74)	95 (100)	1	
1 parent salarié	46 (37,70)	76 (62,30)	122 (100)	1,79	(0,95-3,38)
Parents chômeurs	21 (44,68)	26 (55,32)	47 (100)	2,50	(1,10-5,73) *(p=0,01)*
***Taille de la fratrie***					
1 à 2	39 (26,00)	111 (74,00)	150 (100)	1	
3 à 4	36 (40,90)	52 (59,10)	88 (100)	1,97	(1,08-3,59) *(p=0,01)*
5 et plus	16 (61,54)	10 (38,46)	26 (100)	4,5	

#### Facteurs auxquels sont exposés les enfants

La promiscuité est positivement associée aux cas de tuberculose: le risque de tuberculose est trois fois plus élevé chez les enfants vivant dans la promiscuité (IC_95%_ 1,8-5,57 (p'10^-5^)). Le tabagisme passif est faiblement associé aux cas. En outre, la notion de contage est associée significativement aux cas: les enfants vivants avec des tousseurs présentent un risque plus important (OR=2,04, (IC95% 1,11-3,76) (p=0,01)). La cohabitation avec des personnes sous traitement anti-tuberculeux constitue un risque potentiel par rapport à l'absence de contage (OR=4,71, (IC_95%_ 1,76-12,7 (p=0,003))). Enfin, une comorbidité a été définie comme la présence et l'effet d´un ou de plusieurs troubles associés à un trouble ou à une maladie primaire. L’étude montre le faible taux d'enfants tuberculeux n'ayant pas de comorbidité et l'importance de la malnutrition parmi toutes les autres comorbidités (OR=6 (0,52-9,13)). Les cas font plus de décès et de séquelles que les témoins.

## Discussion

### Limite de l’étude

Le nombre de cas et de témoins étudiés ne constituent pas un échantillon représentatif comme dans toutes les études qui se rapportent à des séries hospitalières. La présente étude ne prétend pas pouvoir élucider tous les problèmes concernant les facteurs associés à la tuberculose de l'enfant. Néanmoins, ce travail nous a permis de mettre en exergue des points importants concernant ce thème. Sur les aspects cliniques, l'absence de preuve bactériologique, c´est-à-dire la certitude diagnostique peut limiter l'intérêt de cette étude. Cependant, il est unanimement reconnu que la preuve bactériologique est difficile. Le diagnostic repose souvent sur les faisceaux d'arguments anamnestiques, cliniques et paracliniques de présomption [9]. En plus, chez l'enfant et le nourrisson en particulier, l'absence des examens de certitude ne doit pas faire retarder le diagnostic de tuberculose: tout retard fait courir le risque de dissémination miliaire ou méningée [14], cause de 39% de décès dans notre série.

### Aspects cliniques

#### Principaux signes cliniques

Selon la littérature, 20 à 60% des cas de tuberculose chez l'enfant peuvent n'avoir aucune manifestation clinique [15]. Dans notre série, aucun enfant n'a été asymptomatique. Par ailleurs, la proportion des symptômes concorde plus ou moins avec les formes cliniques retrouvées. Pour les trois premières formes (pulmonaire (24%), méningée (23%) et primo-infection (22%)), les symptômes comme la toux (37,36%), les convulsions (24%), l'amaigrissement (35%) prédominent. Ainsi, il existe une certaine spécificité des symptômes par rapport aux formes cliniques de la tuberculose chez l'enfant à Madagascar.

#### L’âge

Selon des études antérieures, les enfants tuberculeux sont âgés entre 0 à 59 mois [16–18]. Dans notre série, les enfants âgés de 7 à 59 mois sont les plus susceptibles d'attraper la tuberculose avec un risque de 1,10 à 1,16 tandis que les enfants âgés de 60 mois et plus en sont épargnés. La littérature justifie cette situation par le rôle joué par le problème d'allaitement maternel rencontré dans le premier groupe d’âge, car moins de la moitié des enfants sont allaités au sein conformément aux directives de l'OMS à Madagascar [19]. En effet, les mères allaitantes subissent différentes contraintes. Mais encore, à partir du 6^ème^ mois, âge durant lequel l'allaitement maternel n'est plus exclusif, l'enfant change de régime, qui, souvent n'est pas adapté à ses besoins. Il existe aussi un manque de counseling par les donneurs de soins manquant de connaissance et de compétences; les pratiques d'alimentation appropriées ne sont pas mises en œuvre [20]. La malnutrition s'installe alors pour favoriser la survenue de la tuberculose. Par ailleurs, il faut noter pour les cas des 0 à 6 mois que les risques sont souvent peu importants malgré l'exclusivité non respectée de l'allaitement car les anticorps venant de la mère semblent avoir un rôle non négligeable dans la protection du nourrisson contre l'infection, mais le risque de contamination reste le même dans les deux tranches d’âge [21]. La diminution du nombre des cas à partir de 60 mois peut s'expliquer par le fait qu’à cet âge, l'enfant peut s'adapter plus ou moins au régime adulte et son état immunitaire semble plus développé pour résister au BK.

Au total, vues les associations retrouvées non significatives et les résultats des analyses croisées dans notre étude, on ne peut qualifier l’âge de facteur de risque que lorsqu’ il s'associe à un autre facteur comme le problème nutritionnel.

### Genre et état nutritionnel

Parmi les cas, le genre masculin prédomine. Ce résultat rejoint celui d'El Harim au Maroc en 2007 (54,2%) [16] et celui de Mabiala au Congo (53%) en 2008 [22]. Ceci peut être rattaché au fait que le genre masculin est le plus touché par le problème nutritionnel (53%). Par contre, des ouvrages évoquent la vulnérabilité du sexe féminin à la malnutrition [20]. Mais généralement selon la littérature, il n'y a pas de différence significative suivant le genre [23].

Parmi les cas, 26,37% seulement sont eutrophiques et 53,85% malnutris sévères contre 62% et 75% respectivement dans 2 études subsahariennes [18, 22], et 22% dans une étude au Maghreb [16]. Parmi les témoins, 22% sont eutrophiques et 19,1% seulement sévèrement malnutris. Plus précisément, l'effectif des cas augmente avec l'altération de l’état nutritionnel et l'effectif des témoins diminue. Comme mentionné, la malnutrition touche plus le genre masculin en général [21]; ce qui n'est pas valable pour la malnutrition modérée car 33,33% seulement contre 67,66% chez le genre féminin. Suivant une autre étude [24], l'hypothèse que nous pouvons avancer est que dans certaines circonstances (maladies bénignes, poussée dentaire, etc..) les filles semblent souvent plus anorexiques et perdent du poids facilement [20].

Peu de cas le de kwashiorkor ont été trouvés. Aussi dans notre étude, la majorité des enfants tuberculeux malnutris sont marastiques. Ce résultat concorde avec les données des études antérieures [16, 17]. Il est donc juste de dire qu'il existe une interaction entre la malnutrition et la survenue de la tuberculose chez l'enfant.

### Etat vaccinal et les facteurs socio-économiques

Le risque d'attraper la tuberculose est quatre fois plus élevé chez l'enfant non vacciné. Malgré le statut vaccinal de la population d’étude, les cas de tuberculose chez les vaccinés ne sont pas nuls. Nous avons aussi remarqué que les cas sont moins vaccinés (71%) que les témoins (91,2%), l’écart étant assez énorme (20%). Suivant le pourcentage de 23% de méningites tuberculeuses (36% dans la littérature), la vaccination par le BCG (avec ce taux de 84,5%) réduirait la survenue de cette forme extra-pulmonaire avec plus de 65% environ de pouvoir protecteur. Par contre, le pouvoir protecteur à 56% dans la littérature contre les tuberculoses patentes de l'enfant [25] semble être surestimé d'après notre étude: si nous tenons compte du taux de vaccination à 71% des cas, nous avons trouvé 74,4% de formes patentes (au lieu de 44% dans la littérature). Ceci s'explique par le fait que même chez l'enfant vacciné [26, 27], l'existence d'un contage massif et répété pourrait compromettre la protection conférée par le BCG qui, à ce moment-là, serait dépassé.

Nous remarquons aussi que l'absence de vaccination a des rapports avec les facteurs socio-économiques (mères illettrées, parents chômeurs et la taille élevée de la fratrie), la tendance allant vers moins de vaccination. La mère plus instruite saurait mieux soigner et mieux nourrir son enfant. Elle a également plus de chance d’être salariée et moins de risque de connaître une situation de précarité l'empêchant de s'occuper de son enfant [28]. Par ailleurs, le fait de dire que la mère qui travaille n'a pas le temps de s'occuper de son enfant ne semble pas justifié.

D'après les résultats de notre étude, l'association est forte pour une taille de la fratrie à partir de 5. Claudine Pirus en 2011 en France affirme que 34% de couples ayant plus de 4 enfants sont les plus pauvres [29]. La promiscuité qui en découle peut expliquer l'augmentation de l'effectif des cas contre la diminution nette de l'effectif des témoins dans notre étude, selon aussi une étude canadienne [30].

### Contage tuberculeux

Selon l’étude de Mabiala en 2007 [22], la notion de contage est présente dans 62% des cas, dans 48% des cas dans celle de Raobijaona [31], et dans 40,53% des cas dans notre étude. Nos résultats autorisent à dire que la présence d'une personne sous traitement antituberculeux dans la famille expose à deux fois plus de risque que la présence d'un tousseur chronique. En outre, toute toux n'est pas tuberculose et puisque la durée de l’évolution de la tuberculose du contaminateur avant le début de son traitement n’étant pas connue, il laisse à supposer la possible existence d'une durée de contamination plus ou moins longue [32].

### Tabagisme passif

Suivant de nombreuses études réalisées sur ce sujet, telles que celle de Bates et celle d'Amara, le rapport existant entre le tabagisme passif de l'enfant et la survenue de tuberculose s'avère bien connu [33, 34]. Dans notre série, le tabagisme passif est présent chez 58% des cas et chez 46,24% des témoins, soit 1,62 fois plus de risque par rapport aux non-exposés. Cette association faible permet de dire que le tabagisme passif ne joue pas un rôle primordial dans la survenue de tuberculose chez l'enfant.

### Décès

L'association possible de la tuberculose avec la malnutrition ou une autre maladie sous-jacente pourrait en expliquer le taux élevé. Les études de Tchokoteu en 1990 [18] et M'pemba en 2008 au Brazzaville [35] confortent cette hypothèse.

## Conclusion

Le présent travail démontre que la survenue de la tuberculose chez l'enfant repose sur de nombreux facteurs qui s'intriquent le plus souvent et dont la plupart sont déjà décrits dans la littérature. Leur évaluation à l'heure actuelle permet de constater peu de changement, tant sur leur fréquence que sur leur importance pour être susceptible de modifier les actions déjà menées. Nous avons constaté une amélioration de l’état nutritionnel et une nette augmentation du taux de vaccination. Mais les conditions socioéconomiques de la majorité de la population malgache n'auraient cessé d’être bouleversées depuis la crise politique de 2009, avec des impacts sur la santé des enfants. Les enfants sont exposés à la promiscuité, aux problèmes nutritionnels et à la tuberculose qui sera de diagnostic difficile, soit par faute de moyens, soit suite à des problèmes d'interprétation des examens de présomption.
